# Concealed semantic and episodic autobiographical memory electrified

**DOI:** 10.3389/fnhum.2012.00354

**Published:** 2013-01-24

**Authors:** Giorgio Ganis, Haline E. Schendan

**Affiliations:** ^1^School of Psychology, Cognition Institute, University of PlymouthPlymouth, UK; ^2^Massachusetts General Hospital, Athinoula A. Martinos Center for Biomedical ImagingCharlestown, MA, USA; ^3^Department of Radiology, Harvard Medical SchoolBoston, MA, USA

**Keywords:** concealed information, deception, deception detection, ERPs (event-related potentials), semantic memory, episodic memory

## Abstract

Electrophysiology-based concealed information tests (CIT) try to determine whether somebody possesses concealed information about a crime-related item (probe) by comparing event-related potentials (ERPs) between this item and comparison items (irrelevants). Although the broader field is sometimes referred to as “memory detection,” little attention has been paid to the precise type of underlying memory involved. This study begins addressing this issue by examining the key distinction between semantic and episodic memory in the autobiographical domain within a CIT paradigm. This study also addresses the issue of whether multiple repetitions of the items over the course of the session habituate the brain responses. Participants were tested in a 3-stimulus CIT with semantic autobiographical probes (their own date of birth) and episodic autobiographical probes (a secret date learned just before the study). Results dissociated these two memory conditions on several ERP components. Semantic probes elicited a smaller frontal N2 than episodic probes, consistent with the idea that the frontal N2 decreases with greater pre-existing knowledge about the item. Likewise, semantic probes elicited a smaller central N400 than episodic probes. Semantic probes also elicited a larger P3b than episodic probes because of their richer meaning. In contrast, episodic probes elicited a larger late positive complex (LPC) than semantic probes, because of the recent episodic memory associated with them. All these ERPs showed a difference between probes and irrelevants in both memory conditions, except for the N400, which showed a difference only in the semantic condition. Finally, although repetition affected the ERPs, it did not reduce the difference between probes and irrelevants. These findings show that the type of memory associated with a probe has both theoretical and practical importance for CIT research.

## Introduction

The logic of concealed information tests (CIT) is that stimuli that are known or familiar to people should elicit a different response relative to comparable stimuli that are new (Lykken, 1959). Such tests could have various forensic applications, for example, to determine whether a person who denies having information about certain crime details or certain sensitive information actually possesses such information. CITs have been studied for many decades using several dependent variables, including long-standing, peripheral psychophysiological measures (Ben-Shakhar and Elaad, 2003) and, more recently, electrophysiological (event-related potential, ERP) (Rosenfeld et al., 1988, 1991, 2008; Farwell and Donchin, 1991; Allen et al., 1992) and hemodynamic ones (functional magnetic resonance imaging, fMRI) (Langleben et al., 2002; Phan et al., 2005; Christ et al., 2009; Nose et al., 2009; Ganis et al., 2011).

ERP-based CITs have garnered increased attention lately due to several advantages (e.g., Rosenfeld et al., 2008). (1) They have shown high accuracy rates reliably in detecting concealed information in mock crime scenario paradigms, at least in the laboratory conditions tested. (2) They are relatively inexpensive to implement. (3) The data can be acquired relatively quickly by using a few recording sites on the head. However, the underlying neural mechanisms are largely undetermined. A critical, understudied issue in the field is that people can learn and remember information about an event in many ways.

For example, memory theories distinguish between semantic and episodic memory (Tulving, 1972), and different brain systems have been implicated in each. Episodic memory depends on mediotemporal lobe structures, especially the hippocampus, whereas semantic memory does so much less, if at all, and depends on association cortex, such as anterior temporal cortex (Vargha-Khadem et al., 1997; Schmolck et al., 2002; Eichenbaum et al., 2007; Patterson et al., 2007; Bayley et al., 2008). That different brain systems support episodic and semantic memory raises the important issue that the brain signatures should differ when concealed information revealed on a CIT relies to different degrees on episodic vs. semantic memory. For example, evidence from developmental amnesia patients, who have hippocampal damage and impaired episodic but spared semantic memory, suggests that even residual hippocampal function (despite 50% volume loss or more) is necessary and sufficient to support relative sparing of the ability to imagine false events (Maguire et al., 2010), which is a necessary episodic memory ability for effective deception; neural signatures of such hippocampal activity would thus be expected to be greater for a CIT based on episodic memory relative to one based on semantic memory.

Indeed, the episodic-semantic distinction extends also to the kind of autobiographical memory typically tested in CITs (e.g., Martinelli et al., 2012), the focus of this paper. There are episodic and semantic forms of autobiographical memory. An episodic memory encompasses concrete and unique details associated with distinct events that were experienced by a person in a specific spatiotemporal context and, critically, becomes an episodic autobiographical memory (EAM) when this memory also refers to the self in relation to that context (Tulving, 2002). For example, details about a specific experience that happened at a certain time and place that caught one by surprise. In contrast, semantic autobiographical memory (SAM) encompasses personal information, including general knowledge of personal facts not associated with a specific time and place of acquisition (e.g., “my name is Pat” or “my birthday is December 5th”) and non-specific events, including both repeated and extended events (e.g., schema and script knowledge about “birthdays” not associated with any specific time and place, such as that birthdays are fun and involve friends and family) (Schank and Abelson, 1977). Studies in neurological patients confirm this distinction. For example, amnesic patient K.C. (Tulving, 1993) could report semantic knowledge, such as his own date of birth, but not any autobiographical episodic information (e.g., autobiographical details about any specific birthday). An important question is whether autobiographical probes associated with high semantic vs. episodic memory are associated with different neural processes in the context of a CIT, as would be predicted by neurocognitive studies of these two types of memories (e.g., Tulving et al., 1988; Martinelli et al., 2012). This question also has applied relevance because it could provide information about the brain signatures of these different types of memories that can inform how to maximize detecting concealed information in specific cases. It is important to note that, although there may be distinct neural systems supporting EAM and SAM (e.g., Martinelli et al., 2012), most information in real life is often associated with both EAM and SAM, though with different relative strengths. Note that, for simplicity, in the rest of the paper we will often omit the attribute “autobiographical” and refer simply to semantic and episodic memory.

The main previous ERP study that addressed a related question with an explicitly applied focus is one by Rosenfeld et al. (2006). “High-impact” and “low-impact” probes were compared that differed in semantic and episodic memory content. The high-impact probe was the participant's name, whereas the low-impact probe was the experimenter's name (i.e., “JULIE”). The ERP differences between high-impact probes and a set of random control names (referred to as “irrelevants” in the CIT literature) were much larger than those between the low-impact probes and the irrelevants (i.e., the CIT effect was larger for high than low-impact probes). However, important issues about this finding need to be resolved. First, the same low-impact probe was used for all participants (i.e., the experimenter's name was always “JULIE”). This raises the concern that there could be something intrinsically special, and consistently so across participants, about this name (e.g., frequency, length, associations). This confound was not present for the high-impact probes, as they varied across participants. Furthermore, it is unclear whether the female name used for everybody in the low-impact condition might have been processed differently by male and female participants (i.e., Julie is a female name), as well as individuals (i.e., different people named Julie that each one knows), increasing variability in the results. The ERPs were also recorded from only three sites, limiting assessment of spatial distribution differences between conditions. Finally, the study examined only the P3, leaving it open what effects other ERPs might show, such as the centroparietal N400 marker of semantic memory (Kutas and Federmeier, 2011) or the parietal late positive complex (LPC) associated with episodic recollection (Rugg and Curran, 2007). We would argue that the better way to describe the high- and low-impact probes is in terms of how they activate different kinds of memory. For example, both probes activate semantic and episodic memory, but in different ways for the participant's name (“high-impact”) and the experimenter's name (“low-impact”). Specifically, the participant's name could activate semantic memory more automatically than episodic memory, on average, because people are overlearned experts at responding to their own name, whereas most episodic memories associated with their name would be remote and many would be highly similar and so not distinctly memorable, such as people calling their name, potentially resulting in a lot of interference for recalling associated episodic memories and making them effortful to activate (Soderlund et al., 2012). Thus, semantic memory would be exceptionally automatic for the participant's name, consistent with evidence for a large auditory N400 for one's own name relative to other proper names and no evidence for a posterior LPC effect, suggesting little difference in episodic memory for one's own name and other proper names (Muller and Kutas, 1996). However, by telling subjects that the experimenter's name is “Julie,” subjects acquire a recent episodic memory, which is less effortful to activate than the more remote memories associated with one's own name (Soderlund et al., 2012), predicting a larger LPC for the experimenter's than participant's name, but this has not yet been examined to date. In summary, we would suggest that in the study by Rosenfeld et al. (2006), the participant's name would predominantly activate SAM, whereas the experimenter's name would predominantly activate recent EAM, but such ideas have not yet been systematically addressed.

Thus, the first goal of the current study was to address the question of concealed information based on different types of memory more directly while getting around the limitations in the previous work. First, comparable stimuli without a gender component were used for the semantic (the participant's date of birth) and episodic (a “secret” date given to the participant just before the study) autobiographical memory conditions. Second, all probes and irrelevants varied by person, eliminating any systematic biases in the group average. Third, 32 recording sites were used, enabling potential scalp distribution differences in the ERPs elicited by the two conditions to be determined. Fourth, and related to the previous point, not only the P3 but also other ERPs were evaluated, including the frontal N2, the N400, and the LPC.

A second important issue that has not been addressed systematically in the ERP literature is the effect of stimulus repetition. Because of the relatively low signal-to-noise ratio achievable with all behavioral and psychophysiological measures employed, the typical CIT paradigm averages several tens of trials in which probes and irrelevants repeat many times. Differences between probes and irrelevants using psychophysiological measures, such as skin conductance, decrease rapidly with stimulus repetition because of habituation (e.g., Ben-Shakhar et al., 1975; Ben-Shakhar and Elaad, 2002). However, the same effect may not be present with ERP measures because they may tap into different mechanisms. Furthermore, potential differences between semantic and episodic probes may change over the course of the experimental session. For example, repeated presentation will reactivate semantic and/or episodic memories associated with a probe but do less so if at all for irrelevants, since no distinct semantic or episodic information is available about them. This could result in a difference between probes and irrelevants that becomes larger over time, as ERP repetition effects can be greater for meaningful than meaningless items (Schendan and Maher, 2009; Voss et al., 2010). Another possibility is that repetition of the probes might alter the activation of the semantic and/or episodic memory underlying each. For example, the episodic probe might develop increasing associations with the experimental context, resulting in development of semantic memory (Gratton et al., 2009). This might reduce the N400 (which is smaller when semantic memory activates more successfully) (Voss et al., 2010; Voss and Federmeier, 2011), thereby reducing differences between semantic and episodic probes. On the other hand, all stimuli, including the semantic probe, might develop additional episodic memories with each exposure in the experiment, resulting in additional episodic memories that might increase the LPC (which is larger for more episodic memory), thereby also reducing differences between semantic and episodic probes and associated CIT effects.

The key idea in classical CIT theories is that probes will generate an orienting response associated with, for example, increased skin conductance (e.g., Sokolov, 1963; Gati and Ben-Shakhar, 1990). Although these theories may be adequate to explain autonomic nervous system findings, they cover only a subset of the central nervous system processes engaged by a probe during the CIT (relative to irrelevants) and implicitly assume that probes activate only one kind of memory. However, in the framework described here, semantic and episodic probes may be associated with different neural processes.

Current theories of memory predict that semantic probes would primarily activate semantic memories stored in the neocortex and indexed by ERPs such as the N400 and P3b, whereas episodic probes would primarily activate episodic memory stored in mediotemporal and linked cortical structures, indexed by late parietal potentials, such as the LPC (Paller and Kutas, 1992; Rugg et al., 1998; Dien et al., 2004; Voss and Paller, 2006). In practice, most stimuli are associated with both semantic and episodic memories, and so they would elicit some combination of these effects. For the stimuli in this study, one's date of birth is associated with strong SAM, activating meaning-related processes about oneself in semantic memory but also activating episodic memories incidentally (e.g., events during a specific birthday party, although this may be reduced by providing only the day and month of each date). Prior to the experiment, the birth date is also associated with relatively remote episodic memories of birthday events and other experiences involving one's birth date, such as filling out applications (e.g., for jobs, insurance, taxes). In addition, as the birth date is repeatedly experienced over the course of the experiment, each of these experiences may be encoded as a new (1) episodic memory (Paller and Wagner, 2002) and/or (2) constructed memory that combines new and old (i.e., due to incidental recollection of various birth date memories) episodic elements as well as semantic memory (Hassabis and Maguire, 2009).

New, recent episodic memory encoding can also occur for a different date with no semantic or episodic memory associated with it before the experiment, such as the secret date. Importantly, while multiple trace theory proposes that the hippocampus supports all episodic memories, regardless of how long ago they were encoded (Nadel et al., 2000), some evidence suggests that different parts of the hippocampus support more recent vs. remote episodic memory (Kesner and Hunsaker, 2010; Mankin et al., 2012). Further, between 3 days and 3 months after the learning episode, episodic memories may become semantic by increasing connectivity between cortical areas while decreasing connectivity with the hippocampus (Harand et al., 2012), and a study comparing episodic memories for events ranging in time from very recent (3–14 days old) to very remote (10 years old) found evidence that the hippocampus and the EAM cortical network are integrated more strongly for recent than remote memories (Soderlund et al., 2012). Consequently, more remote memories require more strategic top-down processes in prefrontal cortex for them to be retrieved than do more recent memories. This predicts that ERP effects related to EAM will be greater for the secret date, which involves very recent episodic memory, than the birth date, which involves mostly much more remote episodic memory.

On the other hand, the secret date is minimally meaningful (i.e., low in semantic memory) relative to the birth date. Repeated experiences with any date could potentially begin to construct new semantic memory about that date (Curran et al., 2002; Gratton et al., 2009), but the ability to do so would be minimal because little meaningful information is provided about any dates within the experiment. Notably, the information that the probe is a secret date to be kept concealed during the experiment is meaningful and could lead to learning this as new semantic memory due to repeated experiences with it; knowledge and semantic memory typically require multiple experiences to acquire (Glisky and Schacter, 1987; Verfaellie and Cermak, 1994). Another important way that all these semantic and episodic memory processes could affect the CIT is by inducing standard oddball effects thought to be related to ongoing contextual updating processes in working memory (Kutas et al., 1977; Donchin and Coles, 1988; Dien et al., 2004; Polich, 2007). This could result in a larger P3b to the probes than irrelevants. Further, the P3b to probe conditions could differ as a function of the relative combination of associated semantic and episodic memory. In sum, the birth date potentially activates a combination of high semantic memory and remote episodic memory for multiple birthdays related events, whereas the secret date potentially activates a combination of low semantic memory and recent episodic memory for a single event. Despite reflecting a combination of memory influences, the birthdate and secret date provide an interesting and important starting point for assessing the role of semantic and episodic memory in CITs.

The focus of this paper is on the frontal N2, N400, P3, and LPC components. The frontal N2 is important because recent studies suggest that concealed information in CITs modulate this component with visual (Gamer and Berti, 2010) and auditory stimuli (Matsuda et al., 2009), with probes eliciting a larger frontal N2 than irrelevants. This would be predicted by orienting reflex theory (Ben-Shakhar and Elaad, 2003), as the probe is more meaningful than the irrelevants and occurs infrequently (it is “novel” within the local stimulus sequence). If the frontal N2 reflects primarily an orienting reflex to meaningful information, the N2 should be larger for (1) probes and targets than irrelevants, and (2) semantic autobiographical information, such as one's date of birth, relative to recently acquired episodic information, such as a random (secret) date seen just before the study. However, the frontal N2 is known to be modulated by other variables as well, including the extent to which a stimulus matches to memory (e.g., Folstein and Van Petten, 2008; Folstein et al., 2008): the less a stimulus matches memory, the larger the N2. The precise type of memory involved is usually not specified, but knowledge (e.g., of an object category) and working memory have been mainly studied so far. Thus, an alternative prediction can be made based on the idea that match to knowledge is relevant for N2 modulation. The numbers and month abbreviations used as stimuli will activate knowledge about numbers and months, respectively. This predicts that the N2 will be larger to the irrelevants (minimal memory: people have minimal knowledge about the numbers in random dates that have no task relevance) than a meaningful item (e.g., birth date with rich semantic and remote episodic memories). In addition, depending upon how much new memory is encoded for the episodic item (e.g., a “secret” probe date will be associated with new episodic memory and possibly new knowledge induced by repetition within the experimental context), the N2 to this item may be in-between that to irrelevants and the semantic item.

The centroparietal N400 is larger when an item activates semantic memory less relative to more successfully (Kutas and Federmeier, 2011). Although people know the numbers and month abbreviations used to denote dates, an arbitrary date is not very rich in meaning. In contrast, one's birth date is personally meaningful because it is rich in SAM. This predicts that the N400 will be larger for irrelevant dates than the semantic item (birthdate). In addition, as with the frontal N2, depending upon the extent to which new semantic memory is encoded for the episodic item, its N400 may be in-between that to irrelevants and the semantic item. However, the N2, which merely requires new knowledge to be acquired, may be more sensitive to the memory manipulations in this experiment than the N400, which requires the more demanding encoding of a meaningful representation. After all, the episodic manipulation can induce new knowledge to be learned, but this new information is minimal in meaning, and meaningful representations would typically require a stronger induction event than that used in this experiment (Gratton et al., 2009). For example, acquisition of category knowledge with minimal associated meaning modulates a frontocentral N2 but not necessarily the N400 (Folstein et al., 2008). The N400 may thus show little or no difference between irrelevants and episodic items, instead differing primarily between irrelevants and semantic items.

The effect of concealed information on the P3 has been investigated in numerous ERP studies (e.g., Rosenfeld et al., 1988; Allen et al., 1992; Rosenfeld et al., 2004), but almost all used fewer than five recording sites and so differences between the spatial distribution of the P3 in the different conditions may have been missed. Indeed, the P3 is a family of components, and what has usually been referred to as P3 in previous studies is most likely an instance of the P3b, which has been dissociated from the P3a (Dien et al., 2004; Polich, 2007; Verleger, 2008). The P3b is known to be modulated by many factors, including the subjective probability of items in a perceived category, the complexity of the task and stimuli, and stimulus value (e.g., Johnson, 1986, 1993). We predicted that the P3b to probes would be larger than to irrelevants, replicating previous findings (e.g., Rosenfeld et al., 2004). Further, the semantic probes might elicit a larger P3b than the episodic probes in part because they were the only items associated with strong semantic memory and so they may stand out more in the stream of irrelevants, which are associated only with episodic information acquired during the study.

Finally, the LPC is typically larger during tasks that entail the reactivation of episodic memories (Rugg and Curran, 2007) and so we expected the LPC to be larger to probes, for which episodic memories have been clearly associated, than to irrelevants, for which episodic memory is minimal, and larger to probes in the episodic than semantic condition.

## Materials and methods

### Subjects

Twenty-five naïve healthy volunteers (18 females, between 18 and 35 years of age, mean = 21, *SD* = 3.5 average age: *z* years), recruited from the University of Plymouth (UoP), took part in for course credit. Data from eight participants were excluded due to excessive artifacts (7) or failure to carry out the task as instructed (1). Participants had normal or corrected vision, and no history of neurological or psychiatric disease. All procedures were approved by the UoP Ethics Board.

### Stimuli

**Figure 1:**
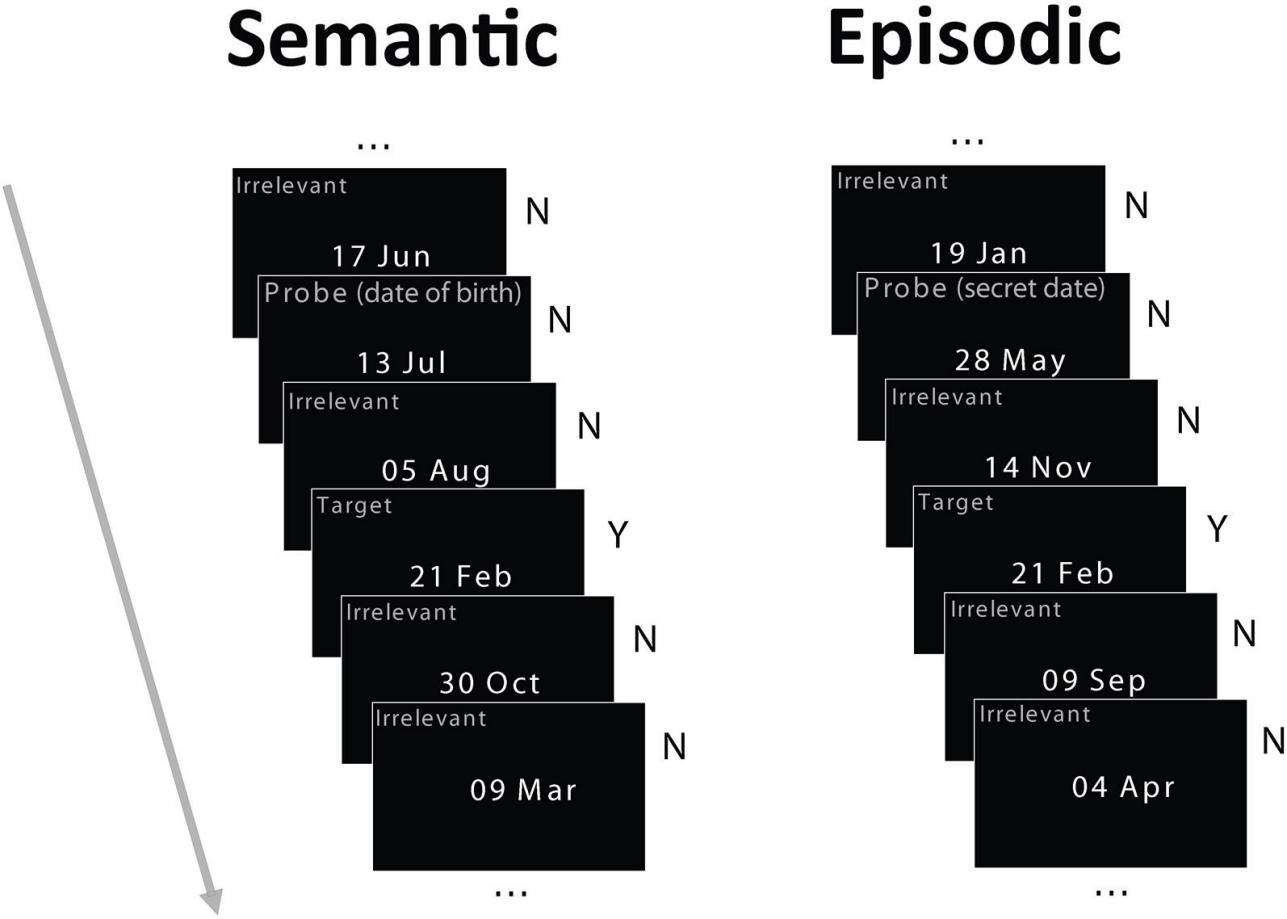
**Schematic of the experimental paradigm.** Participants were tested in two memory conditions in separate blocks: semantic autobiographical and episodic autobiographical. In both conditions, they saw four irrelevant dates, randomly intermixed with a target date and a probe date. In the semantic autobiographical condition, the probe was the participant's date of birth. In the episodic autobiographical condition, it was a secret date in an envelope each participant opened just before the study. Participants reported whether they possessed associated memories for any of the dates, responding honestly to both the irrelevant dates (by pressing the “no” key) and the target date (by pressing the “yes” key), but lying about their birth date or secret date (by pressing the “no” key). Note: Item type labels in the figure shown for illustration only and did not appear on the stimuli.

The stimuli were dates in the format “day month” (e.g., 15 Apr, Figure 1) commonly used by our European participants, subtending about 3 × 2° of visual angle. Three types of dates were used in each condition: irrelevants, probe, and target. During the week preceding the study, at the same time detailed and demographics and health questionnaires were administered, participants were asked over the phone to provide their own date of birth (only the day and month were required) and a list of other important dates (dates of birth of close relatives and friends, anniversaries and so on), so that a set of irrelevant dates could be generated for each participant that excludes these personally important dates. For the semantic autobiographical condition, the probe was the birth date of each participant. For the episodic autobiographical condition, the probe was a date that differed from all other dates used in the study and was not on the participant's list of important dates. The irrelevant dates used for the episodic and semantic conditions were always different. Irrelevant dates never shared the day or the month of the probe or target dates, and they were never famous dates. Furthermore, the target never shared the day or month of the probe.

### Procedure

Before beginning the EEG setup, participants were shown a target date and then were unexpectedly taken into an adjacent fire refuge area by an assistant and the experimenter and they were given an envelope containing their “secret” date. Next, the experimenter left the room, and participants were told by the assistant to open the envelope and to memorize the secret date contained in it, ensuring not to do anything that could reveal they knew this date to the experimenter. Participants were also told that this was their own secret date, different from everyone else's, and that they should keep the note it was written on in their pocket or purse. After setting up the EEG cap and electrodes, participants were seated on a comfortable chair in front of a computer screen (about 114 cm away) in a dark room. Two conditions were administered in separate blocks, the semantic and episodic conditions, with order counterbalanced across participants. In the semantic condition, the probe date was the individual's birth date whereas, in the episodic condition, it was the “secret date.” This secret date varied by participant to match the between-participant variability of the date of birth. In both semantic and episodic conditions, participants were instructed to deny possessing any memory for the probe date (birth date or secret date, respectively) throughout the session by giving a deceptive “no” response. They were also instructed to give an honest “yes” response about knowing the target date. Thus, participants had to report honestly whether they knew each date, but they had to lie about the probe date. In sum, participants responded honestly to both the target (pressing “yes”) and the irrelevants (pressing “no”) but deceptively to the probe (pressing “no”). Participants responded by pressing one of two buttons with the index and middle finger of their dominant hand. They were instructed to respond as fast as possible without sacrificing accuracy. Each item was presented for 800 ms with an inter-trial interval of 3000 ms. In each condition, each item (four irrelevants, one probe, and one target) was presented 35 times in a pseudo-random order for a total of 210 trials. The constraints on the pseudo-random sequence were that a probe and a target could never appear in temporally adjacent trials, and any individual irrelevant could only repeat for a maximum of three times in the sequence. The same abstract sequence (i.e., the sequence of irrelevant, probe and target types of items) was used for the two conditions to eliminate potential differences due to sequence statistics. Each condition was split into two blocks of ~7 min each, to test the effect of stimulus repetition. There was a short practice session (10 trials) before the experimental trials. Finally, at the end of the study, participants were asked to recall the target and the secret dates and indicate if they had any pre-experiment memory associated with any of the other dates. Since there was 100% recall accuracy in all cases, the recall data were not further analyzed.

### Electrophysiological data acquisition

The electroencephalogram (EEG) was sampled at 250 Hz from Ag/AgCl electrodes (gain = 20,000, bandpass filtering = 0.01–100 Hz). EEG data were collected from 32 electrodes arranged in a geodesic array (Figure 3) and additional electrodes placed below the right eye referenced to left mastoid to monitor eye blinks, on the tip of the nose, and the right mastoid, all of which were referenced to the left mastoid. Note that in this configuration, Fz is just posterior to site 27, Cz coincides with site 28, and Pz is just posterior to site 29. Horizontal eye movements were monitored using two electrodes placed on the outer canthi of the right and left eyes, referenced to each other. Electrode impedance was below 5 kΩ for all channels.

### Analyses

Performance measures were submitted to ANOVAs with three factors: item type (average of irrelevants; probe; target), memory condition (semantic and episodic), and repetition (first and second half). To ensure participants carried out the task, follow-up ANOVAs also contrasted targets with irrelevants and targets with probes. However, the main comparison of interest for each memory condition was between probes and irrelevants because the same response (“no”) was associated with both. As this comparison was the main focus of this experiment, and targets received a different response (“yes”) from all other items, confounding their comparison with other items, ERP analyses focus only on an item factor that include probes and irrelevants; note, preliminary analyses that included ERPs to the targets confirms expected target P3b effects. In the following, significant differences between probes and irrelevants (in the behavioral or ERP data) will be referred to as the CIT effect.

#### Response times

Response times (RTs) and accuracy rates were analyzed in the omnibus ANOVA and planned comparisons.

#### ERPs

ERPs were averaged off-line for an epoch of 1000 ms, including a 100 ms baseline. Trials affected by blinks, eye movements, muscle activity or amplifier blocking were rejected off-line. An average of 31 artifact-free trials per item type per participant went into the analyses (*MIN* = 16, *SD* = 4.2). A One-Way ANOVA showed no differences in the number of trials across conditions (including both repetitions), *F*_(5, 85)_ = 1.05, *p* > 0.1, η^2^ = 0.06. Data were analyzed unfiltered but shown filtered at low-pass 30 Hz in the figures. Repeated measures ANOVAs on the mean amplitude of the average ERPs assessed the effects of item type and condition on the N2, N400, P3, and LPC components. The time windows used for the main analyses centered arounds the mean peak latency of the N2 (250–350 ms), the N400 (350–500 ms), the P3 (400–600 ms), and the LPC (750–900 ms). To assess the overall pattern of results, a “lateral” ANOVA assessed lateral sites (13 pairs, see electrode montage in Figure 3) using factors of Item Type (probes vs. irrelevants), Site, and Hemisphere. A second, “midline” ANOVA assessed the midline sites (six electrodes) using factors of Item Type and Site.

Planned focal analyses were also conducted at frontal sites 1 and 2 for the N2, central site 28 (Cz) for the N400, and parietal site 30 for the P3b and LPC, where these components were maximal. These analyses compared (1) probes and irrelevants (i.e., the CIT effect) in both memory conditions, since we predicted differences between probes and irrelevants in both cases, and (2) probes between the two conditions, since we predicted differences between the semantic and episodic probes. Note that we did not carry out amplitude-latency analyses on the P3b because the overlapping N400 made it difficult to determine P3b peak latency in single participants. The focal analysis was carried out on the mean amplitude data within the time windows used in the main analyses. At focal sites, onset of the CIT effect (i.e., probes vs. irrelevants) and the difference between semantic and episodic probes was determined. For the N2 and N400, 25 ms time windows were used, between 100 ms and 350 ms, and 300 and 550 ms, respectively. A paired *t*-test between the conditions of interest was carried out on each time window until a significant difference was found in three successive time windows. The time window preceding the first significant time window was used as an estimate of the onset time of the effect. For the P3, the same logic was used with 25 ms time windows between 200 and 600 ms.

## Results

### Behavior

**Figure 2:**
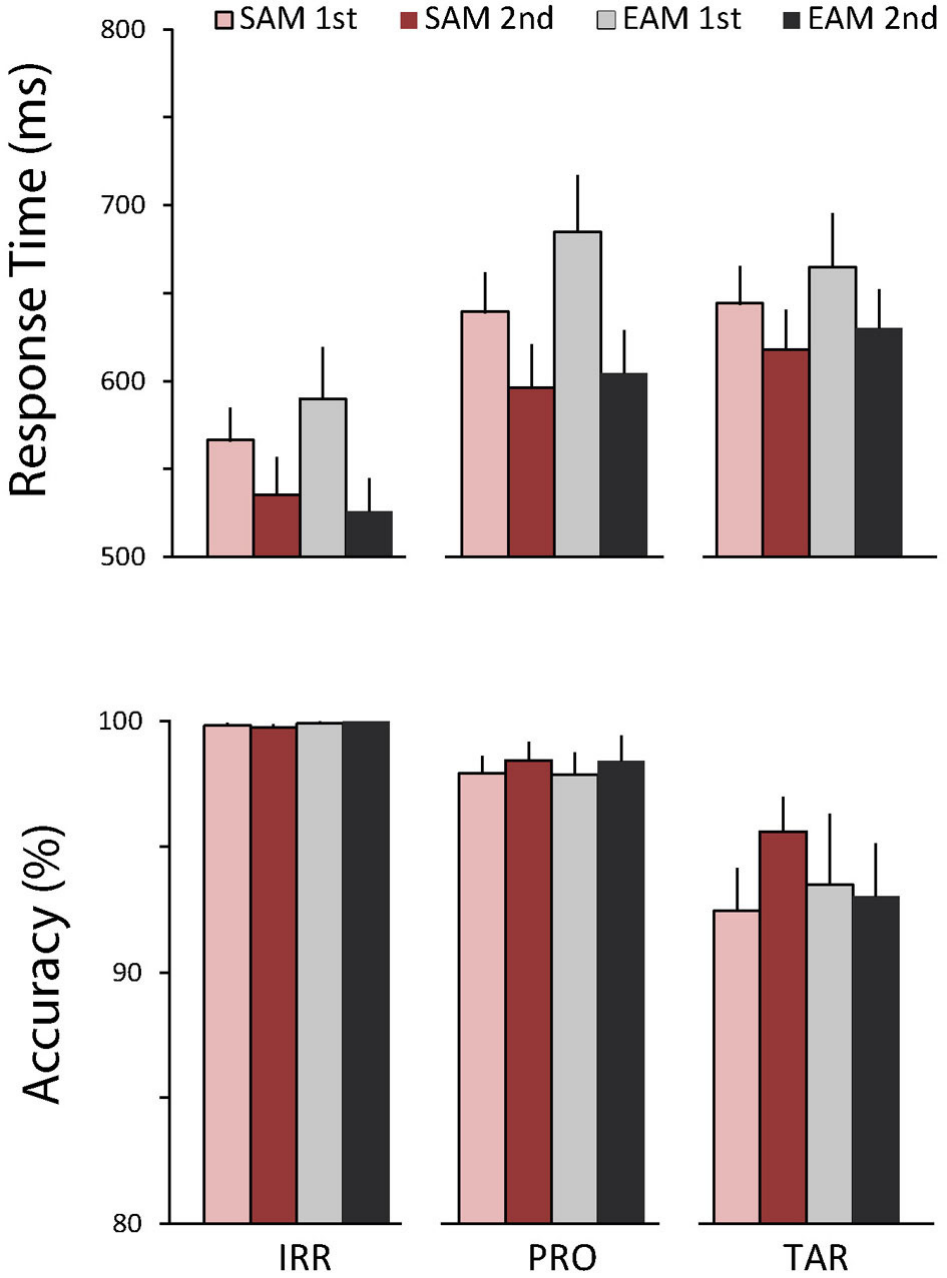
**Behavioral results.** Top, average response times (RTs) to irrelevants, probes, and targets in the semantic (red bars) and episodic (gray bars) autobiographical conditions during the first (light bars) and second (dark bars) repetition. Bottom, accuracy for the same conditions. Error bars depict 1 SEM.

Figure 2 shows the behavioral results. RTs varied by item type, *F*_(1, 16)_ = 54.09, *p* < 0.001, η^2^ = 0.77. Furthermore, RTs were faster in the second than first half of each memory condition block, *F*_(1, 16)_ = 21.49, *p* < 0.001, η^2^ = 0.57, and this repetition effect was modulated by item type, *F*_(2, 32)_ = 4.73, *p* < 0.05, η^2^ = 0.23. Follow-up analyses to parse this effect compared each item type with the other two. RTs were slower to probes than irrelevants, *F*_(1, 16)_ = 73.90, *p* < 0.001, η^2^ = 0.82, and both RTs were faster in the second than first half, *F*_(1, 16)_ = 25.32, *p* < 0.001, η^2^ = 0.61, but the repetition effect tended to be marginally larger in the episodic than semantic condition, *F*_(1, 16)_ = 3.68, *p* = 0.07, η^2^ = 0.19. Similarly, RTs were also slower to targets than irrelevants, *F*_(1, 16)_ = 85.84, *p* < 0.001, η^2^ = 0.84, and both RTs were faster in the second than first half, *F*_(1, 16)_ = 12.35, *p* < 0.005, η^2^ = 0.44, but the repetition effect tended to be marginally larger in the episodic than semantic condition, *F*_(1, 16)_ = 3.22, *p* = 0.09, η^2^ = 0.17. In contrast, RTs to targets and probes were similar, and both RTs were faster in the second than first half, *F*_(1, 16)_ = 24.11, *p* < 0.001, η^2^ = 0.60, but probes were slower than targets in the first half, whereas the opposite held in the second half, *F*_(1, 16)_ = 8.30, *p* < 0.05, η^2^ = 0.34. Accuracy showed only a main effect of item type, *F*_(1, 16)_ = 15.36, *p* < 0.001, η^2^ = 0.49. Follow-up analyses revealed that accuracy was lower for targets than both irrelevants, *F*_(1, 16)_ = 17.11, *p* < 0.001, η^2^ = 0.52, and probes, *F*_(1, 16)_ = 13.36, *p* < 0.005, η^2^ = 0.46. Accuracy was also lower for probes than irrelevants, *F*_(1, 16)_ = 11.67, *p* < 0.005, η^2^ = 0.42. Notably, there were no significant main effects of memory on RTs and accuracy and no significant repetition effects on accuracy.

### Event-related potentials (ERPs)

**Figure 3:**
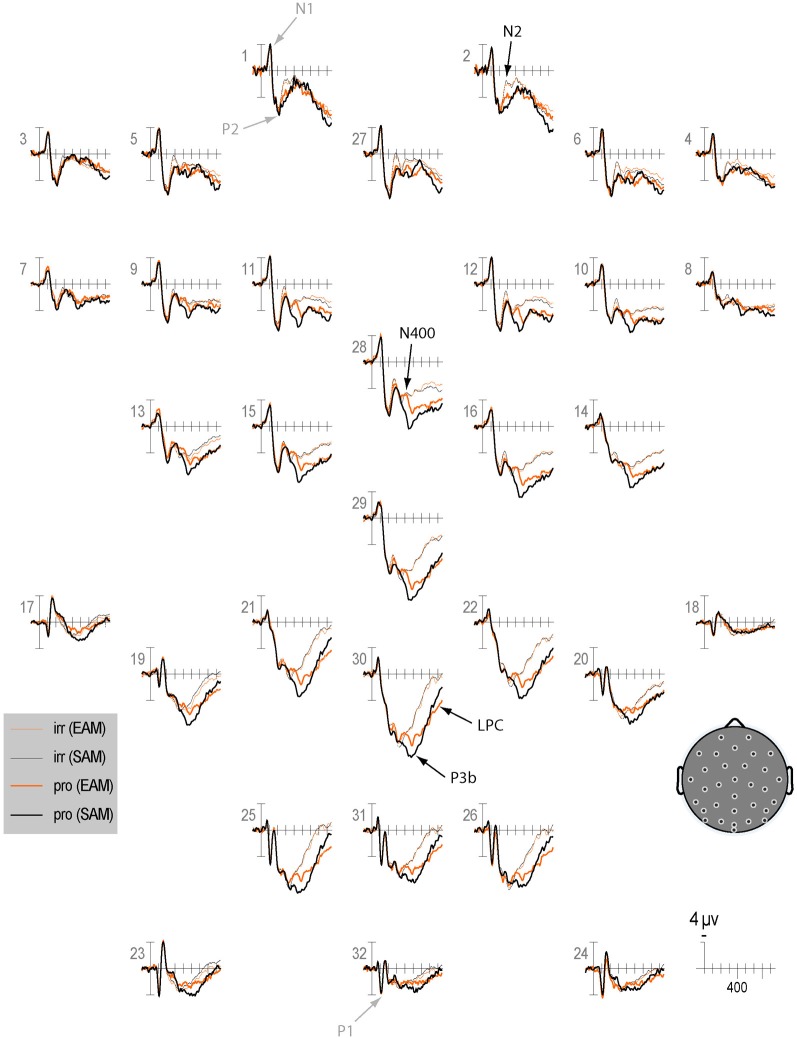
**Grand average ERPs elicited by irrelevants (thin solid lines) and probes (thick solid lines) in the semantic (black lines) and episodic (red lines) autobiographical conditions.** ERPs are plotted between-100 and 900 ms (at all scalp recording sites). ERPs are shown negative up and referenced to the average of the left and right mastoids. A diagram with the location of the recording sites is shown on the bottom right.

**Figure 4:**
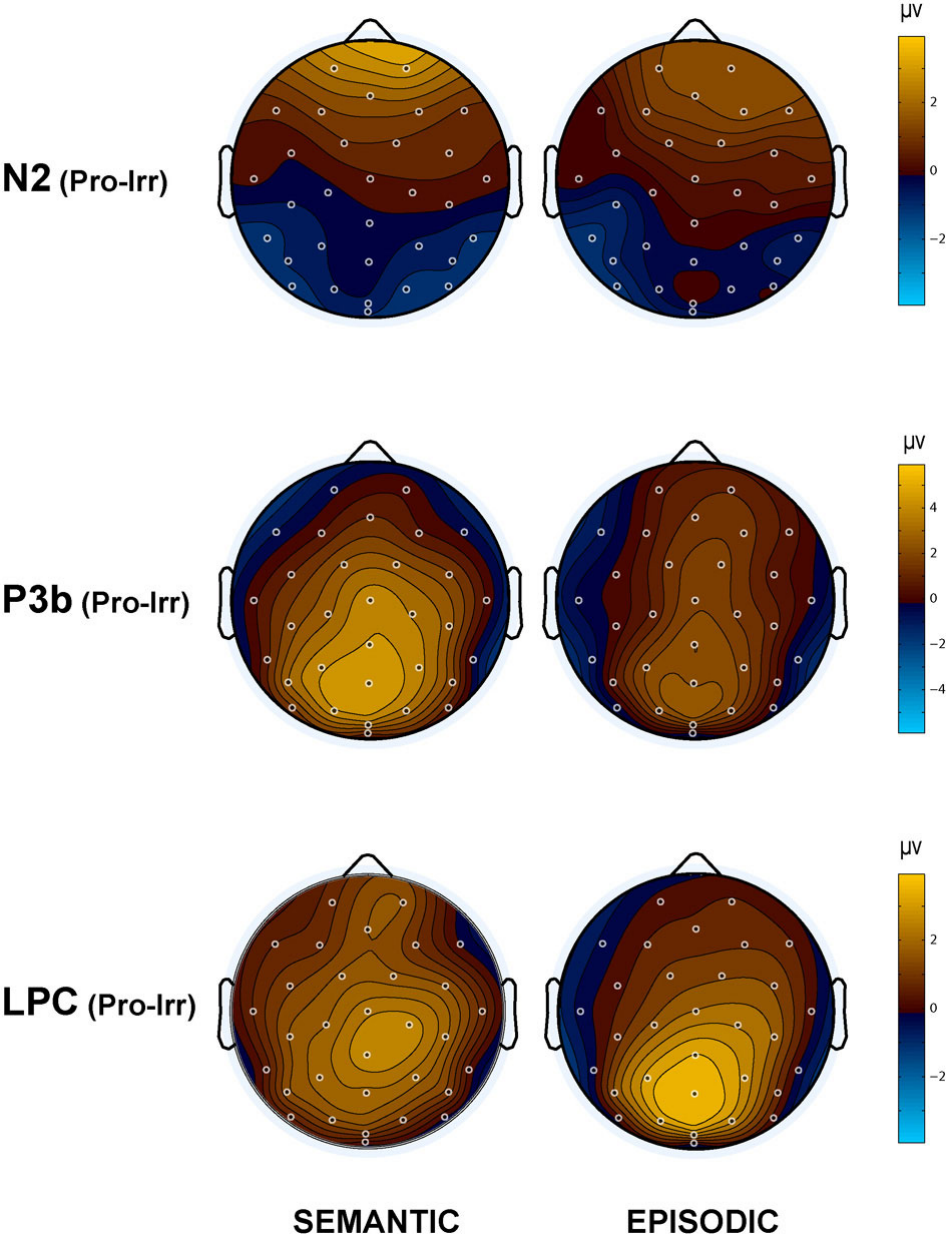
**Topographic maps of the ERP difference between probes and irrelevants for (top) the N2 (250–350 ms), (middle) P3b (400–600 ms), and (bottom) LPC (750–900 ms) in the semantic (left column) and episodic (right column) autobiographical conditions.** An N400 map is not shown because there was no CIT effect in the episodic condition, and the P3b map for the semantic condition captures the N400 as a positive difference at Cz (28); thus, the centroparietal distribution in the P3b time period of the semantic condition reflects the combination of the overlapping central maximum of the N400 CIT effect and the parietal maximum of the P3b CIT effect. Note, the voltage scale is not the same for all topographic maps.

**Figure 5:**
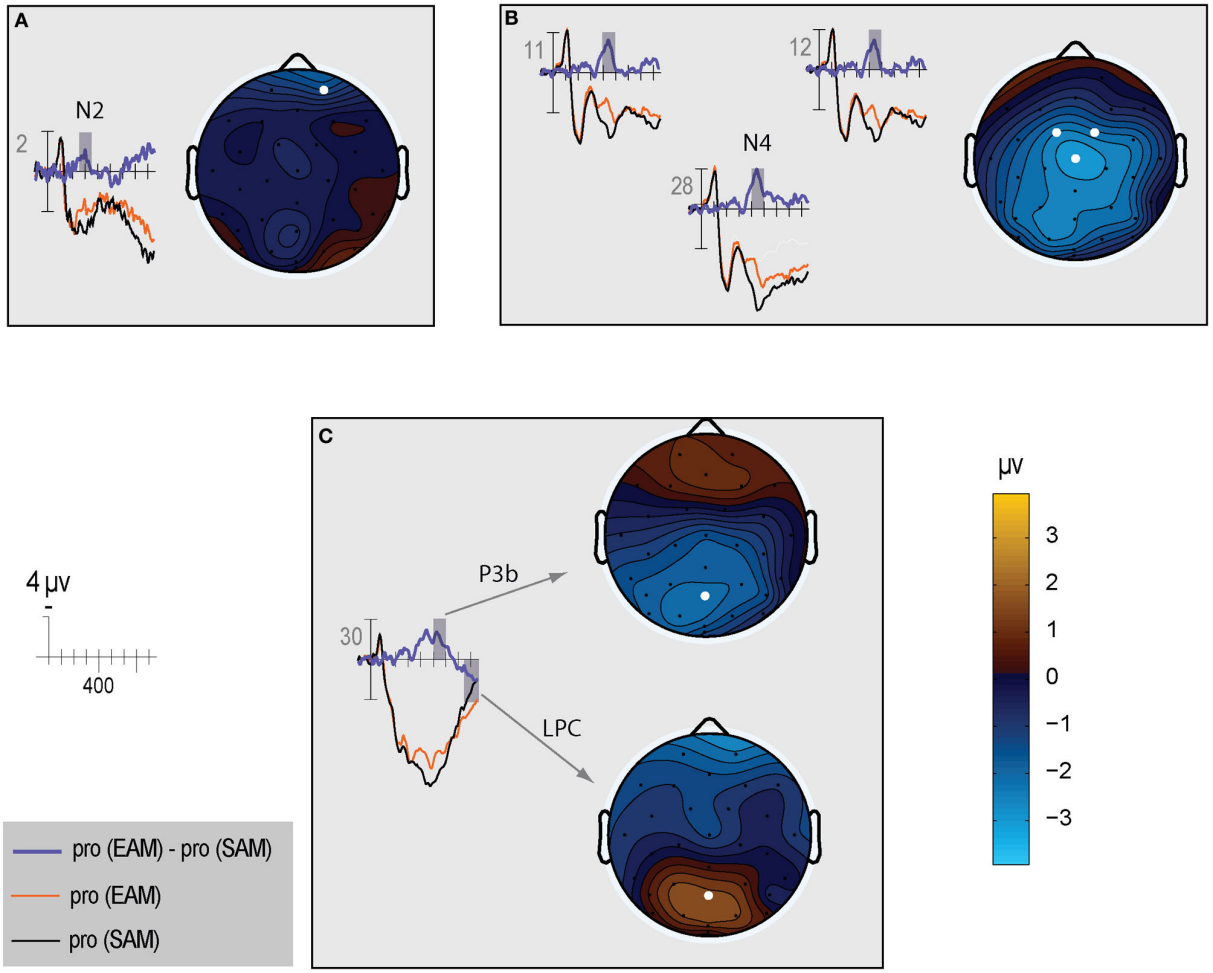
**The left side of each panel shows the ERP time course for episodic probes (thin orange line), semantic probes (thin black line), and difference between episodic and semantic probes (thick blue line).** The right side of each panel shows a topographic map for the difference wave shown on the left: **(A)** N2; **(B)** N400; **(C)** P3b and LPC.

**Figure 6:**
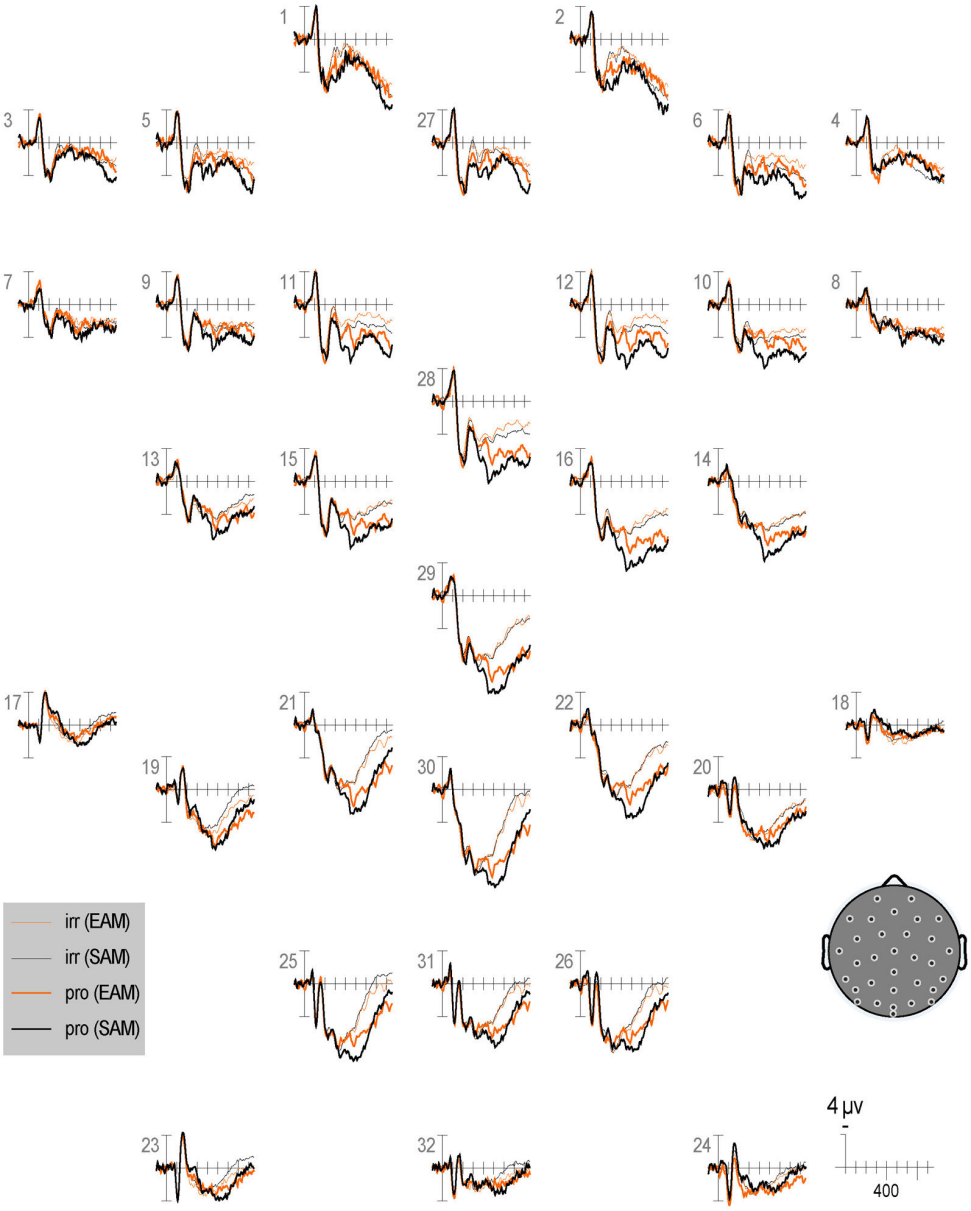
**Grand average ERPs elicited by irrelevants (thin solid lines), probes (thick solid lines) in the semantic (black lines) and episodic (red lines) autobiographical conditions in the first half of trials.** ERPs are plotted between 100 and 900 ms (at all scalp recording sites). ERPs are shown negative up and referenced to the average of the left and right mastoids. A diagram with the location of the recording sites is shown on the bottom right.

**Figure 7:**
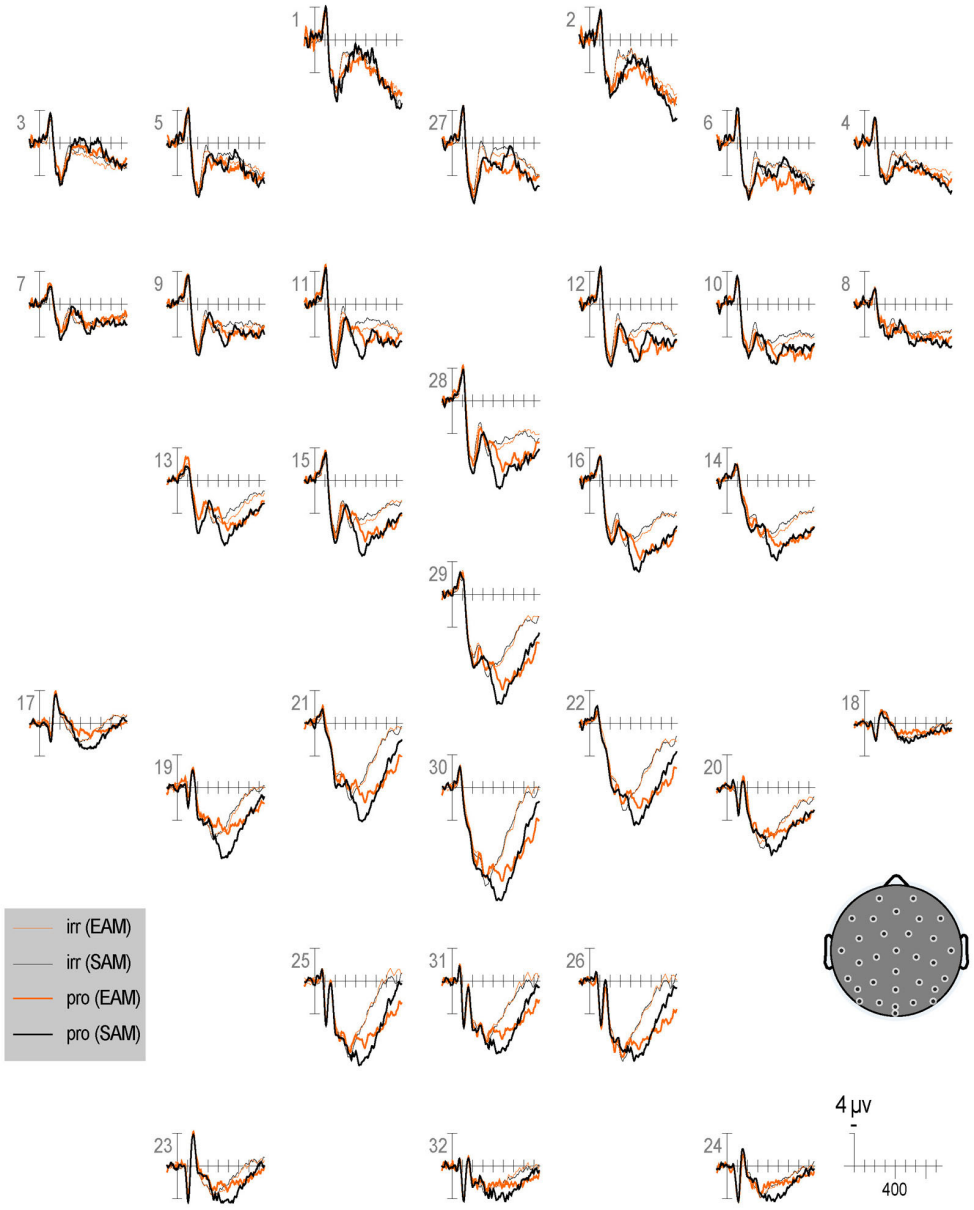
**Grand average ERPs elicited by irrelevants (thin solid lines), probes (thick solid lines) in the semantic (black lines) and episodic (red lines) autobiographical conditions in the second half of trials.** ERPs are plotted between 100 and 900 ms (at all scalp recording sites). ERPs are shown negative up and referenced to the average of the left and right mastoids. A diagram with the location of the recording sites is shown on the bottom right.

Qualitatively, the ERP waveform showed an occipitotemporal P1 and a corresponding anterior N1, followed by a frontocentral P2 and N2, and a centroparietal N400, P3b, and LPC (Figures 3–7). Four main differences between items and memory conditions are evident in the ERPs. The first difference is on the N2, maximal at frontal sites between 250 and 350 ms (Figures 3, 4 and 5A). Second, a clear N400 component overlapping the first part of the P3b is present in the episodic condition, maximal at central sites, and to a lesser extent in the semantic condition (Figures 3 and 5B). The third difference is on the P3b, maximal at centroparietal sites between 400 and 600 ms (Figures 4 and 5C). Fourth, LPC differences appear later at the same sites, lasting until the end of the epoch (Figures 4 and 5C). Omnibus statistics are shown in Tables 1–3 and described below.

**Table 1:** **Results of the omnibus lateral (Lat) and midline (Mid) ANOVAs for the N2 (probe vs. irrelevants, 250–350 ms)**.

	**N2**							
	**Lat**				**Mid**			
	***F***	***p***	**η^2^**		***F***	***p***	**η^2^**	
I	3.87	0.07	0.19	^°^	1.68	0.21	0.09	
I × S	**14.14**	**0.00**	**0.47**	^***^	**9.57**	**0.00**	**0.37**	^***^
I × S × H	1.47	0.19	0.08					
R	**6.42**	**0.02**	**0.29**	^*^	**8.78**	**0.01**	**0.35**	^**^
R × S	1.84	0.16	0.10		2.62	0.07	0.14	^°^
R × S × H	**2.16**	**0.03**	**0.12**	^*^				

I, Item; R, Repetition; S, Site; H, Hemisphere.

° p < 0.1;

* p < 0.05;

** p < 0.01;

*** p < 0.001.

#### N2 (250–350 ms) and N400 (350–500 ms)

***N2.*** Omnibus results at lateral and midline sites (Table 1) showed a larger N2 for irrelevants than probes at frontal and frontocentral sites (I × S), and ERPs were more positive during the second than the first half (R, lateral sites, 3.81 vs. 3.31 μV, respectively; midline sites, 5.55 vs. 4.79 μV, respectively, Figures 6 and 7). At lateral sites, repetition effects were maximal at centroparietal sites and larger over the right hemisphere at frontocentral sites, but symmetric or larger over the left hemisphere at more posterior sites (R × S × H).

Planned focal analyses on frontal pair 1 and 2 showed that probes were more positive than irrelevants (4.35 vs. 2.10 μV, respectively), *F*_(1, 16)_ = 40.01, *p* < 0.001, η^2^ = 0.71, and this CIT effect tended to be larger on the right than the left (3.46 vs. 2.99 μV, respectively), *F*_(1, 16)_ = 4.01, *p* = 0.063, η^2^ = 0.20. Importantly, this CIT effect was larger in the semantic than episodic condition, *F*_(1, 16)_ = 5.53, *p* < 0.05, η^2^ = 0.26, due to the probes in the semantic condition being more positive than those in the episodic condition (4.87 vs. 3.83 μV, respectively), *F*_(1, 16)_ = 4.50, *p* < 0.05, η^2^ = 0.22. This result is the opposite of the hypothesis that the N2 CIT effects reflect orienting to novelty but consistent with the alternative hypothesis that the N2 is sensitive to match to knowledge. No repetition effects were significant.

The onset of the CIT effect in the two memory conditions was determined at right frontal site 2, where the differences were largest. Results showed that the CIT effect onset between 200 and 225 ms in the semantic probe condition, and slightly later, between 225 and 250 ms, in the episodic probe condition. A second onset analysis showed that the onset of the difference between probes in the two memory conditions was also between 225 and 250 ms.

***N400.*** The N400 is the only ERP component to show a CIT effect only in the semantic condition. The N400 is smallest for the semantic probe, relative to the episodic probe and all irrelevants, which are indistinguishable from each other (Figure 3). Figure 4 (middle) shows an overall centroparietal scalp distribution between 400 and 600 ms due to the combination of the central CIT effect on the earlier N400 and the parietal CIT effect on the later P3b. Figure 5B shows the memory effect around central sites where the N400 overlaps least with the frontal N2 and parietal P3b, illustrating that the N400 is more negative to episodic than semantic probes and has a central maximum and overall centroparietal scalp distribution, which is characteristic of the N400 index of semantic memory (Kutas and Federmeier, 2011). The omnibus analyses on the N2 and P3b capture the early and late part of the N400, so the focus was on planned focal analyses.

A focal analysis on Cz (site 28) showed that probes were less negative than irrelevants (7.29 vs. 5.16 μV, respectively), *F*_(1, 16)_ = 7.05, *p* < 0.05, η^2^ = 0.31, and this effect was larger in the semantic than episodic condition (3.27 vs. 0.96 μV, respectively), *F*_(1, 16)_ = 6.16, *p* < 0.05, η^2^ = 0.28. A follow-up analysis showed that the difference between probes and irrelevants was only significant in the semantic condition, *t*_(16)_ > 2.35, *p* < 0.05, for both repetitions. ERPs were more positive during the second than first repetition, *F*_(1, 16)_ = 4.58, *p* < 0.05, η^2^ = 0.22, but this effect did not interact with any other factors. Finally, ERPs were more positive during the semantic than episodic conditions, *F*_(1, 16)_ = 8.73, *p* < 0.01, η^2^ = 0.35.

Finally, an onset analysis of the CIT effect in both memory conditions was carried out at Cz. Results showed that the CIT effect onset between 400 and 425 ms in the semantic conditions, whereas it onset between 475 and 500 ms in the episodic condition. A second onset analysis showed that the onset of the difference between probes in the two memory conditions was between 400 and 425 ms.

#### P3b (400–600 ms)

Omnibus results (Table 2) showed a larger P3b for probes than irrelevants at lateral (I, 6.84 vs. 4.30 μV, respectively) and midline sites. This CIT effect was maximal at lateral and midline centroparietal sites (I × S), and lateral results showed that this effect was larger on the right at frontocentral sites but on the left at more posterior sites (I × S × H). Importantly, the difference between probes and irrelevants was larger in the semantic than episodic condition at lateral and midline sites (M × I), and this interaction was largest at centroparietal sites (lateral, M × I × S). ERPs at this time tended to be more positive during the second than the first half (*R*). At lateral sites, this repetition effect was larger on the right at frontal and posterior sites, but symmetrical at central sites, and maximal at right centroparietal sites (R × S × H). The lateral repetition effect was also modulated by item type, as it was larger for probes than irrelevants (I × R × S × H), and by memory type, as it was larger at centroparietal sites in the semantic condition, but at fronto-central sites in the episodic condition (M × R × S).

**Table 2:** **Results of the omnibus lateral (Lat) and midline (Mid) ANOVAs for the P3b (probe vs. irrelevants, 400–600 ms)**.

	**P3b**								
	**Lat**				**Mid**			
	***F***	***p***	**η^2^**		***F***	***p***	**η^2^**	
M × S	1.39	0.25	0.08		**6.33**	**0.02**	**0.28**	^*^
I	**15.17**	**0.00**	**0.49**	^***^	**26.20**	**0.00**	**0.62**	^***^
I × S	**6.64**	**0.00**	**0.29**	^**^	**7.12**	**0.00**	**0.31**	^**^
I × S × H	**3.06**	**0.00**	**0.16**	^**^				
R	2.99	0.10	0.16		4.39	0.05	0.22	^°^
R × H	4.17	0.06	0.21	^°^				
R × S × H	**2.05**	**0.03**	**0.11**	^*^				
M × I	**7.45**	**0.02**	**0.32**	^*^	**6.33**	**0.02**	**0.28**	^*^
M × I × S	**3.49**	**0.04**	**0.18**	^*^	2.33	0.09	0.13	
M × R × S	**3.08**	**0.03**	**0.16**	^*^	2.49	0.07	0.13	
I × R × S × H	**2.66**	**0.01**	**0.14**	^**^				

M, Memory; I, Item; R, Repetition; S, Site; H, Hemisphere.

° p < 0.1;

* p < 0.05;

** p < 0.01;

*** p < 0.001.

Planned focal analyses were conducted at parietal site 30 where the P3b was maximal. Consistent with the omnibus analysis, the P3b was larger for probes than irrelevants, *F*_(1, 16)_ = 38.35, *p* < 0.001, η^2^ = 0.71 (10.77 vs. 7.15 μV, respectively). Importantly, this CIT effect was larger in the semantic than episodic condition, *F*_(1, 16)_ = 5.26, *p* < 0.05, η^2^ = 0.25 (4.80 vs. 2.45 μV, respectively) because the P3b was more positive for the semantic than episodic probes, *F*_(1, 16)_ = 4.53, *p* < 0.05, η^2^ = 0.22 (11.84 vs. 9.7 μV, respectively). There was a non-significant trend for the P3b to be larger during the second than the first half, *F*_(1, 16)_ = 3.56, *p* = 0.08, η^2^ = 0.18, and the CIT effect was numerically larger during the second than the first half, but this interaction of item and repetition was also not significant, *F*_(1, 16)_ = 2.47, *p* = 0.14, η^2^ = 0.13 (4.11 vs. 3.14 μV, respectively). Thus, the CIT effect did not change significantly as a function of repetition (if anything, it became slightly larger).

The onset of the CIT effect in the two memory conditions was determined at parietal site 30 where the differences were largest. Results showed that the CIT effect onset between 375 and 400 ms in the semantic probe condition, and, later, between 450 and 475 ms in the episodic probe condition. The onset of the difference between the probes in the two memory conditions was also analyzed, revealing an onset between 375 and 400 ms.

#### LPC (750–900 ms)

Omnibus analyses (Table 3) showed that the LPC was more positive for probes than irrelevants at lateral (I, 3.77 vs. 2.29 μ V, respectively) and midline sites, and these CIT effects were largest at lateral and midline centroparietal sites (I × S). The lateral ANOVA also revealed that the LPC was larger in the second than first half, and more so over the right hemisphere (R × H), and a significant four-way interaction indicated that the CIT effect was further modulated by repetition and hemisphere (I × R × S × H). In contrast, the midline ANOVA also revealed that the CIT effect was larger in the semantic than episodic condition at centroparietal sites (M × I × S).

**Table 3:** **Results of the omnibus lateral (Lat) and midline (Mid) ANOVAs for the LPC (probe vs. irrelevants, 750–900 ms)**.

	**LPC**							
	**Lat**				**Mid**			
	***F***	***p***	**η^2^**		***F***	***p***	**η^2^**	
M × S	**4.15**	**0.01**	**0.21**	^*^	0.52	0.48	0.03	
I	**31.24**	**0.00**	**0.66**	^***^	**29.80**	**0.00**	**0.65**	^***^
I × S	**4.79**	**0.00**	**0.23**	^**^	**9.09**	**0.00**	**0.36**	^***^
R × H	**4.72**	**0.05**	**0.23**	^*^				
M × I × S	1.57	0.17	0.09		**2.93**	**0.02**	**0.15**	^*^
M × R × S × H	1.73	0.09	0.10	^°^				
I × R × H	3.52	0.08	0.18	^°^				
I × R × S × H	**2.47**	**0.02**	**0.13**	^*^				

M, Memory; I, Item; R, Repetition; S, Site; H, Hemisphere.

° p < 0.1;

* p < 0.05;

** p < 0.01;

*** p < 0.001.

Planned focal analyses conducted at parietal site 30 where the LPC was maximal confirmed that the LPC was more positive for probes than irrelevants, *F*_(1, 16)_ = 30.19, *p* < 0.001, η^2^ = 0.65 (3.79 vs. 0.64 μ V, respectively), and in the episodic than semantic condition, *F*_(1, 16)_ = 4.97, *p* < 0.05, η^2^ = 0.28 (2.70 vs. 1.72 μ V, respectively). Follow-up analyses showed that probes in the episodic condition elicited a larger LPC than probes in the semantic condition *F*_(1, 16)_ = 5.25, *p* < 0.05, η^2^ = 0.25 (4.67 vs. 2.91 μ V, respectively). No repetition effects were significant.

## Discussion

In summary, performance is consistent with previous CIT studies using the 3-stimulus paradigm with speeded responses in that responses for probes and targets are slower and less accurate than for irrelevants (e.g., Gamer et al., 2007; Gamer and Berti, 2010). The present results also provide evidence for repetition priming, as responses to all items are faster in the second than the first half of each memory condition block, on average. ERPs show multiple effects. First, the frontal N2 is larger to irrelevants than both types of probes, and the CIT effect on the N2 starts by 225 ms in the semantic condition but slightly later, by 250 ms, in the episodic condition. Second, semantic and episodic probes begin to be processed differently by 250 ms, and this early effect is maximal at frontal sites, where the N2 is larger for episodic than semantic probes. Third, the N400 shows a CIT effect only in the semantic condition, as a central N400 is smaller for the semantic probe relative to the episodic probe and irrelevants, which resemble each other. Fourth, probes generate a larger P3b than irrelevants, and this CIT effect starts by 400 ms and is larger for semantic than episodic probes. Fifth, episodic probes generate a larger LPC than semantic probes. Sixth, although ERPs became more positive in the second half of the trials, the CIT effect on the P3b remains similar. Next, we discuss these findings in turn.

### Performance

The behavioral results indicate that probes and targets are more difficult to process than irrelevants. The typical explanation for this finding is that both infrequent probes and targets stand out in the stream of irrelevants but require different responses. This creates a conflict that takes some time to resolve (Gamer et al., 2007). Note that, since targets were the only items requiring a “yes” response, the direct comparison between targets and irrelevant is not very informative. The pattern of behavioral effects was the same for both memory conditions. This indicates that the ERP differences between these conditions do not reflect RT or accuracy differences, and suggests that the probes in these two conditions were similar in terms of saliency. The overall repetition effect on the RTs, but the lack of a repetition effect on the difference between probes and irrelevants, indicates that repetition had mostly a generic effect independent of item type.

### Frontal N2 and centroparietal N400: knowledge and semantic memory

#### N2

At least two types of frontal N2 components have been distinguished, a cognitive control N2 and a memory (mis)match N2, whose amplitude is modulated by different factors (Folstein and Van Petten, 2008; Folstein et al., 2008). Concealed information studies have focused on the cognitive control N2 and most used an orienting reflex account. Clearly, the pattern of effects on the frontal N2 found here is not consistent with a simple orienting reflex explanation (Sokolov, 1963). The N2 is largest for the frequent irrelevants which, according to an orienting reflex account, should be the least salient stimuli and so should be associated instead with the smallest N2.

Most previous CIT studies using ERPs have focused exclusively on the P3b component, making it difficult to compare our results with those of the previous literature. More troublesome, the low-pass filtering employed in some studies is so extreme (around 4 Hz in some cases) that any effects on fast changing components like the frontal N2 would be wiped out (Rosenfeld et al., 2006). However, two recent CIT studies examined the effect of the experimental manipulations on components of the N2-family (Matsuda et al., 2009; Gamer and Berti, 2010). The study by Matsuda and collaborators used a 2-stimulus paradigm (i.e., no targets were present), auditory stimuli of an episodic nature (single digits), long interstimulus intervals (22 s) to enable peripheral psychophysiological recordings, and a common average reference montage, making it difficult to compare the results with those of the current study with visual stimuli, fast intertrial intervals, and average mastoid reference. Their findings showed a slightly larger central N2 for probes than irrelevants, which the authors suggest is an N2b reflecting the redirection of attentional resources to salient stimuli (Matsuda et al., 2009). The study by Gamer and Berti (2010) is perhaps more comparable to the present study since it employed visual stimuli, a 3-stimulus paradigm, and a right mastoid reference. This study reported a larger frontal N2 to probes than irrelevants, and attributed such an effect to cognitive control processes required for response monitoring. Such an explanation would predict a larger frontal N2 for probes than irrelevants in the current study as well, but the opposite was found. It is possible that differences in the paradigms could account for this discrepancy: The stimuli differed (playing cards were used in that study compared to dates here), different interstimulus intervals were used (8 s, on average in that study vs. 2 s here), and stimuli were not counterbalanced across participants in the earlier work, leaving open the possibility of item-specific confounds. These differences in the paradigm clearly resulted in ERP differences compared with standard CIT results as, for example, there was no P3b effect. Furthermore, a subsequent CIT study by the same group (Gamer and Berti, 2012) failed to find any N2 effects. Although further work is required to fully characterize the factors that affect the frontal N2 in CIT paradigms, the current study shows that concealed information is not necessarily associated with a larger frontal N2 in CIT paradigms and that the literature is inconsistent.

The pattern of N2 effects suggests that the degree to which an item matches memory, a factor known to modulate frontal N2 amplitude (i.e., larger N2 for memory mismatch), is the key factor modulating the N2 in this study. This interpretation is supported by the finding that the frontal N2 is smaller for the date of birth, the item associated with the most semantic (and remote episodic) memory, followed by the secret date, which is associated with recent episodic memory for the learning experience in which the participant received the envelope with this date (and perhaps some newly acquired semantic memory, e.g., the fact that the date is a secret), and by the irrelevants, which have very little associated semantic or episodic memory. Based on this finding alone, we cannot rule out that this N2 effect reflects both semantic and episodic memory, but prior evidence implicates semantic memory more. The N2 memory match effect has primarily been found when knowledge is manipulated, not in episodic memory experiments. This knowledge is not necessarily semantic (meaning) *per se*, because frontal effects, especially frontopolar ones where the N2 is maximum here, are not always found with semantic manipulations (Ganis and Kutas, 2003; Kutas and Federmeier, 2011). A visual knowledge interpretation is also indicated by evidence that a frontal N3(00) complex from 200 to 500 ms, which includes the memory match N2 as an early component of this waveform, is specific to processing visual images (Barrett and Rugg, 1989, 1990; McPherson and Holcomb, 1999) and modulated according to how successfully visual knowledge is activated for a category decision (e.g., dog, cat, car) (Schendan and Kutas, 2002, 2003; Schendan and Maher, 2009). Finally, it is noteworthy that ERPs in the N2 time window became more positive with repetition, but the effect did not vary by item type. Such an increase over multiple repetitions may reflect accumulation of knowledge with each repetition, as in category learning, which can modulate the frontal N2, the N400, and other ERPs (Curran et al., 2002; Folstein and Van Petten, 2004; Folstein et al., 2008; Scott et al., 2008; Gratton et al., 2009).

#### N400

This interpretation of the N2 is consistent with the modulation of the N400 index of semantic memory, which is clearest at central sites [see site C*z*(28) in Figure 5B] where the N400 overlaps least with the frontal N2 and the parietal P3b. The N400 is more negative for episodic than semantic probes because the amplitude of the N400 is inversely proportional to the amount of semantic memory associated with both linguistic and non-linguistic stimuli (e.g., Kutas and Federmeier, 2011): A secret random date acquired just before the study has little or no semantic memory associated with it, compared to one's birth date, which is by far the most meaningful stimulus. Consequently, meaning activates most successfully for this semantic probe, and its N400 is smallest. In contrast, the N400 is larger to the episodic probe (secret date), but comparably as large to the irrelevants. The similarity between the N400 to the episodic probe and the irrelevants is consistent with the fact that the meanings of all these items are minimal and about the same (i.e., just the meaning of the numbers and months but no other richly meaningful facts). This also indicates that the new information about the secret date acquired before the study did not result in a sufficiently meaningful representation to affect the semantic memory processes underlying the N400. Notably, in contrast, the secret date information did result in new knowledge, such as visual knowledge, as demonstrated by the smaller frontal N2 for the episodic probe relative to the irrelevants. This is consistent with evidence that certain types of newly acquired knowledge result in sensitivity of the frontal N2 (and similar frontal negativities between 200 and 500 ms, e.g., frontal N3 complex, N300, N350, N390 components) to this knowledge (Curran et al., 2002; Ganis and Kutas, 2003; Folstein and Van Petten, 2004, 2008; Folstein et al., 2008; Schendan and Maher, 2009), but additional more richly meaningful information needs to be provided for the centroparietal N400 to become sensitive to newly acquired facts about an item (Gratton et al., 2009). Importantly for deception detection, this means that the N400 shows a CIT effect for semantic autobiographical information, and quite a robust one, but minimal to no CIT effect for episodic autobiographical information.

Previous CIT studies have not reported N400 effects for several reasons. First, the N400 effect is largest at Cz(28) but overlaps to some extent the P3b spatiotemporally at this site and parietal sites. A number of CIT studies have used only the three midline sites Pz, Cz, and Fz, or reported data only for those sites (e.g., Rosenfeld et al., 1988, 2004, 2006; Gamer and Berti, 2010), and so may have missed N400 effects or analyzed them as part of the P3b effects. Second, as mentioned in the context of the frontal N2, extreme low-pass filtering to enhance slow components like the P3b might have spuriously reduced effects on faster-varying components such as the N400 (e.g., Rosenfeld et al., 2006). A third reason is suggested by the present finding of a CIT effect on the N400 only in the semantic condition. Episodic stimuli do not show a CIT effect because, in the present work and many previous studies, they do not have sufficiently rich semantic memory representations to produce a CIT effect on the N400.

From a memory perspective, it is necessary to consider the alternative that the frontal N2 and centroparietal N400 effects reflect instead episodic memory. Indeed, frontal negativity between 100 and 300 ms (during the N2) does show memory effects, being more negative for new than old items during recognition tasks (Tsivilis et al., 2001), but the interpretation of such repetition effects, and similar ones on the N400, is controversial (Rugg and Curran, 2007) and, if anything, points to knowledge, conceptual memory, and semantic memory (Paller et al., 2007; Voss et al., 2010; Voss and Federmeier, 2011). These issues have been discussed in detail in the debate about whether an N400-like component, which sometimes appears to have a more frontal (and so labeled “FN400”) than centroparietal distribution, reflects episodic familiarity or conceptual implicit memory (due to activation of meaning representations) (Paller et al., 2007; Rugg and Curran, 2007; Voss and Federmeier, 2011). While this debate is beyond the scope of this paper, it is relevant to consider whether the frontal N2 or N400 pattern might reflect episodic familiarity. We suggest that familiarity can't simply or easily explain the N400. First, one might argue that the semantic probe (one's birthday) has more lifetime familiarity than the episodic probe (an arbitrary date with no other meaning) because episodic memories set up prior to the EEG recording are numerous (albeit more remote) for one's birthday but only singular (albeit more recent) for the episodic probe. These pre-existing episodic memories for one's birthday therefore reduce the N400 (or N2) for the semantic more than the episodic probe. Second, both semantic and episodic probes are equally as familiar in terms of exposure during EEG recording (i.e., repeated the same number of times), which is the typical way that episodic familiarity is defined experimentally. This predicts no difference between semantic and episodic probes, in contrast to the clear memory effects observed. Third, connectivity between the hippocampus and neocortex is stronger for recent episodic memory, which is the kind primarily associated with the episodic probe, relative to remote episodic memory, which is only associated with the semantic probe (Soderlund et al., 2012). Such differential hippocampal-cortical linkages would predict a greater reduction in episodic memory-related cortical activity for the episodic than semantic probe, thereby resulting in a smaller N400 (or N2) for the episodic than semantic probe—the opposite of the observed pattern. Fourth, altogether, these episodic memory considerations would predict a larger N400 for the irrelevants than the episodic probe because the episodic probe was studied beforehand but the irrelevants were not (and so are less familiar), but no evidence was found for any difference between the episodic probe and irrelevants. The parsimonious explanation is that the consolidated semantic memory in the cortex for the semantic probe drives the N400 pattern, as argued here. Consistent with this, the FN400 has been argued to be identical to the N400 and to reflect semantic memory and conceptual implicit memory for repeated items (Paller et al., 2007; Voss et al., 2010; Voss and Federmeier, 2011). Note, as all items repeated here many times, conceptual priming (due to conceptual implicit memory) could explain the N400 pattern, not only semantic memory (Renoult and Debruille, 2011). The N400 shows robust modulation with conceptual priming, being smaller for repeated than new meaningful items (Paller et al., 2007). Conceptual priming would be greater for the meaningful semantic probe than the minimally meaningful episodic probe (Voss et al., 2010), consistent with the observed pattern.

Together, these findings suggest that the N2 and N400, as highly sensitive markers of knowledge and semantic memory, respectively, could potentially be used for detecting concealed information, but only if the type of memory is considered carefully and the concealed information that one is trying to detect is stored in the brain systems for knowledge and semantic memory. In contrast, if the goal is to detect episodic memory, then later brain potentials, like the P3b and LPC, may be more suitable markers. In most realistic cases, in which the probes are associated with both semantic and episodic memory, both types of markers should be considered.

#### P3b

The main prior study that addressed an issue similar to the one addressed here is the one by Rosenfeld and Collaborators (2006). The relevant finding from that study is that the P3b difference between probes and irrelevants was much smaller for low-impact probes (the recently learned experimenter's name) than high-impact probes (a participant's name). In fact, the difference between probes and irrelevants in the low-impact condition was close to zero. Like that study, we found that semantic probes elicit a larger P3b than episodic ones: The CIT effect is larger in the semantic than episodic condition. Even so, at least in the analysis within a fixed P3b window, episodic probes show a sizeable CIT effect on the P3b. One possible explanation for this discrepancy is that, in the prior study, the low-impact probes were incidentally learned, even though they were encountered numerous times in the experiment. In the current study, participants were explicitly told that the (episodic) probe was a secret date that they had to lie about. This constitutes intentional encoding, which results in greater episodic memory than incidental encoding and likely also increases the saliency of such a date (Hyde and Jenkins, 1973; Craik and Tulving, 1975; Kellogg et al., 1982). Given the intentional nature of deception, intentional study would also be expected to transfer more appropriately to the intentional retrieval situation of the CIT paradigm than incidental study (Tulving and Thomson, 1973; Morris et al., 1977). Since the stimulus sequences used in the two memory conditions were identical, this finding confirms that the P3b is modulated by the type of memory triggered by the probe, not just by context updating taking place in working memory (Johnson, 1986, 1993). Importantly, the CIT effect on the P3b did not become smaller with repetition, but rather, tended to become larger. This indicates that the duration of the test is not a major issue in P3-based CITs, and the benefit of longer ERP sessions with more trials may not be cancelled by habituation effects, as usually seen with electrodermal measures (Ben-Shakhar et al., 1975). Future work will have to determine whether the CIT effect on the P3b is constant for even longer sessions that may be required in the field. It is noteworthy that our results may underestimate the size of the P3b in the episodic condition during its initial phase when it overlaps the N400 at central sites due to the opposing polarities of these ERPs (Figures 3–5), but not afterwards around the P3b peak and thereafter from 500 to 600 ms.

The P3b pattern bolsters the interpretation of the earlier frontal N2 and centroparietal N400 patterns in terms of knowledge and semantic memory. Both the present findings and the Rosenfeld et al. (2006) study indicate that the CIT effect on the P3b is larger for semantic items (e.g., your own birth date and name, respectively) relative to items that are less meaningful or about which one has less knowledge (e.g., a secret date, irrelevants). Likewise, in experiments on semantic memory using an object categorization task, a parietal P3b-like component, peaking around 600 ms, is more positive for objects categorized more than less successfully (Schendan and Kutas, 2002, 2003; Schendan and Maher, 2009). Consequently, in the present study, the P3b is larger for the semantic than episodic probe possibly because subjects more successfully identify the semantic probe as their birthdate relative to the episodic probe as the secret date and discriminate the semantic better than the episodic probe from the irrelevant dates in order to generate a deceptive response; after all, the episodic probe and the irrelevants have minimal to no meaning and so they may be less discriminable from each other in terms of knowledge and semantic memory. Altogether, the N2, N400, and P3b all point to the importance of knowledge and semantic memory for demonstrating a CIT effect on these ERPs.

#### LPC

Even though previous CIT studies have considered episodic memory, no previous ERP study has examined specifically the LPC, which is well established as a marker of conscious recollection from episodic memory (Paller and Kutas, 1992; Rugg and Curran, 2007). In the present study, episodic probes elicit a larger LPC than semantic ones. This finding is consistent with studies of episodic memory in which people decide whether an item is new or old (e.g., Paller et al., 1995). In these studies, a larger LPC is typically found for old items recognized as such (e.g., items with associated episodic knowledge) relative to new items. The parietal distribution and time course of this old/new LPC resembles the LPC memory effect found here. The present LPC finding thus indicates that, when the concealed information is thought to be primarily or predominantly due to episodic memory, then the LPC may be the most robust ERP to examine in CIT paradigms. Intriguingly, the LPC pattern is the only ERP finding that parallels the RT pattern: The LPC is more positive and RTs are slower for episodic than semantic probes, which are slower than irrelevants. However, the LPC starts after the RTs in the episodic condition, on average, suggesting that the recollection process underlying the LPC cannot drive the RT effect.

#### Repetition

Repetition effects should be explored in future ERP work, especially given the sensitivity demonstrated here of RTs and accuracy to this factor. Overall, RTs and ERPs between 250 and 350 ms, 400 and 600 ms, and 750 and 900 ms show repetition effects, but focal analyses on the N2, N400, P3b, and LPC show no repetition effects, perhaps due to insufficient power. Intriguingly, RT repetition effects to all item types (i.e., faster responses in the second than first half) tend to be larger (albeit non-significantly) in the episodic than semantic condition. However, no ERP repetition effect is larger in the episodic than semantic condition, but given the weakness of the RT interaction, power may have been insufficient to detect this also in the ERPs. Nonetheless, it appears that the N2 and N400 are larger for episodic than semantic in the first (Figure 6) more than the second half of trials (Figure 7), whereas the P3b is larger for semantic than episodic, and the LPC is larger for episodic than semantic in the second more than first half. Future ERP studies should manipulate repetition with a greater number of trials and in more subtle ways and evaluate whether repetition modulates the CIT effect on these ERPs and if so, how.

#### Performance and ERPs

RTs, accuracy, and ERP effects differed from each other so it is unclear which ERP effects drive the behavioral effects. Nonetheless, a few points can be made. The LPC starts too late (after 700 ms) to drive RT effects (all faster than 700 ms, on average) and corresponding accuracy of these responses. The P3b (400–600 ms) overlaps the earliest RTs, which are to irrelevants (500–600 ms), and so is also probably too late to influence RTs to irrelevants and even too late to influence RTs to probes much if at all (600–700 ms). The N2 and N400 are thus the ERP markers that are most likely to be responsible for the RTs and corresponding accuracy. Consistent with this, the N2 has long been recognized as having a time course and relationship with RTs consistent with the underlying decision processes driving the RTs during discrimination tasks, such as the CIT, in part because the N2 is early enough to drive the RTs, whereas the P3 is often too late, and N2 latency is related to RTs (Ritter et al., 1979). Likewise, the N3 complex, which includes the (mis)match N2, is related to RTs during category decisions (Philiastides and Sajda, 2006, 2007; Philiastides et al., 2006). Given that both the N2 and N400 show a CIT effect in the semantic condition, the RT CIT effect in this condition could reflect both knowledge and semantic memory processes underlying these ERPs. Given that the N2 but not the N400 shows a CIT effect in both the semantic and episodic conditions, the knowledge processes underlying the N2 but not the N400 drives the CIT effect in the episodic condition. However, it is likely that at least the initial CIT effect on the P3b, which starts within 400 ms in the semantic condition and within 475 ms in the episodic condition, could further influence the RTs. However, the finding that P3b CIT effects end around 650 ms, which is after the response to all items except the episodic probe in the first half of trials suggests that the processes underlying the P3b are unlikely to be the only factor influencing behavior. This highlights the importance of considering the (mis)match frontal N2 and N400 and underlying knowledge and semantic memory processes, respectively, in future CIT studies and for deception detection, in general.

#### Saliency and related factors

Memories can differ in saliency, but how they differ depends upon many factors. Manipulating these factors was beyond the scope of this initial experiment but will be important for future research. We highlight here a few key issues regarding saliency and memory. First, saliency needs to be defined clearly but as yet a good definition is lacking, in general and in the memory field. Saliency has been most clearly defined in the context of selective attention to perceptual information. In particular, saliency is defined operationally by search performance: Items that differ along certain perceptual dimensions from the surrounding context are more salient and can be detected faster than items that do not differ as much from the surrounding context (e.g., a red dot against a background of green distractor dots). Saliency so defined orients selective attention, which can occur in parallel in early visual areas (Treisman, 2006) and, after the initial separation of stimulus information into features, binds these features together into an object representation and searches a scene serially (Wolfe et al., 1989; Treisman, 2006). Depending upon the context and task goals, these computations can inform the selective attention system to attend to salient features (e.g., the red dot) and filter out distractors and less salient features (Kastner and Pinsk, 2004). Selective attention can also be driven endogenously (e.g., by task goals and memory), and the top-down feedback inputs that perform these functions proceed from higher to lower order areas of information processing (Buffalo et al., 2010).

Second, in CIT paradigms usually the various items do not differ perceptually and so “saliency” is driven entirely by stored memory and to its interaction with the details of the CIT paradigm. For example, probes and irrelevants are perceptually identical between conditions (i.e., all strings of two numbers and three letters) and have the same motor demands and so perceptual differences cannot drive saliency here: Saliency is determined primarily by memory. Notably, this dictates that, because memory is stored where it is processed in the cortex (Slotnick and Schacter, 2004; Schendan and Maher, 2009), saliency effects might be observed at the same time as memory differences are computed and/or afterwards when an earlier memory computation influences later cognitive and other memory processes (Moses et al., 2005): Saliency effects can only be observed once the first memory effect has begun. Thus, it is necessary to consider how saliency has been defined in some of the few memory studies that have tried to address its role.

Third, in the memory field, definitions of saliency are based on memory representations, not perception, and differ between memory types: the information encoded in memory and its interactions with the task determine the salience of memory of a particular type (e.g., semantic or episodic), and such memory saliency computations can potentially influence another memory (of the same or a different type) activated simultaneously or later on in stimulus processing (e.g., semantic memory could influence episodic memory). For example, saliency of semantic memory has been defined based on (1) conceptual or perceptual distinctiveness in terms of dominance of meaning (e.g., for “bank,” the dominant meaning is associated with “money,” not “river”) (Rajaram, 1998) or learned statistical regularities of the stimuli (e.g., orthographic frequency) (Rajaram, 1998), respectively, or (2) the representation strength of semantic features (e.g., high visual vs. high motor) (Kellenbach et al., 2002; Koriat and Pearlman-Avnion, 2003). (3) For episodic memory, saliency (or significance) of autobiographical information is based on the personal relevance of the learning episode and is closely related to emotional salience (Westmacott and Moscovitch, 2003), and this helps to preserve episodic memory (Levin et al., 1985) and semantic memory despite brain injury (Westmacott and Moscovitch, 2003). Note, by all these definitions, memory saliency is intrinsically entangled with the memory itself. On this basis, the birth date is higher in the saliency of both semantic and episodic memory than the secret date, predicting larger CIT effects across the entire ERP waveform. However, this was not the case because the LPC, consistent with this ERP as an index of episodic recollection, shows a larger CIT effect for the secret date. This raises the possibility that, for episodic memory, an additional definition of saliency is based on the role of recency (Soderlund et al., 2012). The present findings suggest that episodic memory can be more salient when recent than remote, as the secret date was associated with more recent episodic memory than the birth date. Finally, these considerations and multiple memory systems theory (Schacter and Tulving, 1994), more generally, highlight that saliency can only be defined within a particular type of memory; otherwise, one would be comparing apples and oranges. Saliency for semantic memory is not the same as saliency for episodic memory (e.g., meaning dominance determines salience for semantic memory vs. personal relevance determines salience for episodic memory). Thus, it would be difficult, if not theoretically impossible, to compare directly the saliency of items such as the birth and secret dates. For instance, on the one hand, it is uncommon to ask people explicitly to lie, and so the secret date has a very distinctive and salient episodic memory, and the recency of this memory may also enhance its saliency. On the other hand, the birth date usually has more personally relevant associated episodic memories than the secret date, and so it is very salient as well. In short, memory salience is greater for the birth date based on some definitions and memory types, whereas it is greater for the secret date based on others. Finally, we note that, by definition, semantic memory represents meaning, whereas episodic memory can store information regardless of meaning, as when people recognize non-sense visual patterns (Voss et al., 2011). Accordingly, the birth date is highly meaningful due to activating semantic memory, whereas the secret date is less meaningful due to activating semantic memory less successfully but instead activates episodic memory, due to its recency, more successfully than the birth date. Thus, as we argued, the birth and secret dates differ as a function of their ability to activate semantic or episodic memory, and differ in meaningfulness (as one definition of saliency) only as a function of the extent to which they activate semantic memory.

Fourth, saliency is not a property of an item alone but rather a property of the item in a particular context. For example, the frequent word “table” may be low in saliency when embedded in a list of other frequent words but highly salient in the context of famous names. Furthermore, memory saliency depends on the task at hand and can be modulated by attentional manipulations (Rajaram, 1998), and so the word “table” can become highly salient in the context of other common words when the task requires detecting furniture words that appear infrequently. In the present CIT paradigm, the birth and secret dates occurred (in different blocks) infrequently within a stream of random dates, and they were the only items for which a lie had to be produced, making them highly salient in both conditions. The behavioral results support this and provide an operational definition of saliency for this task, as done in the attention field: faster RTs to probes are taken to reflect higher saliency in the task. Specifically, although RTs differ reliably between probes and irrelevants (documenting that the study had sufficient power to detect such differences), the memory conditions show no evidence of any difference that could be attributed to saliency. Thus, the CIT paradigm and procedures made both types of probes highly salient (being the only items for which a lie had to be produced) so that any residual saliency differences are very small; at most, probes show a non-significant trend to be faster in the semantic than episodic condition in the first block (596 vs. 640 ms). This could be due to the specific content of the memory (birthdate) or to the fact that the semantic probes were the only ones with a strong semantic content in the stream of irrelevants and targets (items with predominantly episodic memory associated with them and minimal semantic memory).

Finally, other reasons why we believe differential saliency was not an issue in the present study include the following. (1) If the birth date is more salient than the secret date, then a saliency-based account of the frontal N2 would predict a larger N2 to probes than irrelevants and a larger N2 for the birth than secret date. This is because, by definition, saliency engages attentional and other cognitive control processes (e.g., response monitoring, depending on the stimulus-response mapping) and these processes are typically associated with a larger N2 (Folstein and Van Petten, 2008). So, counterfactually, a smaller N2 for the date of birth than the secret date implies that date of birth was not more salient than the secret date. The same logic applies to the comparison between probes and irrelevants. Thus, the evidence is at odds with a saliency account and more in tune with a memory matching explanation. (2) Further, the LPC effect should be larger for the birth than the secret date, but the opposite was found, consistent with the idea that memory differences primarily drive the effects. (3) To our knowledge, there has been no systematic P300 CIT work suggesting that a recent memory leads to smaller P300s than one's birth date, unless such information is acquired incidentally (Rosenfeld et al., 2006), which was not the case in our study, and no CIT studies have been conducted in which stimulus saliency was non-circularly defined and its systematic manipulation affected P300 amplitude (or any other ERP) in a way that could easily explain the current findings.

#### Memory task orientation

Memory is task-dependent; task instructions can influence the importance of a particular type of memory for performance and alter the pattern of effects (Richardson-Klavehn and Bjork, 1988). Thus differential activation of semantic and episodic memory by the birth and secret dates may also change the memory orientation of the CIT task to favor semantic vs. episodic memory, respectively. Consider that a seemingly subtle difference in task instructions from categorization (e.g., categorize the object) to recognition (e.g., recognize the item as old or new) alters the cortical networks involved in processing the same item (Schendan and Stern, 2008), consistent with multiple memory system theory (Squire and Zola-Morgan, 1991; Schacter and Tulving, 1994). In the SAM condition, the task requirement to lie about the birthdate likely focuses attention on associated rich semantic memory and remote episodic memories because these memories distinguish the birth date from the target date and irrelevants. Likewise, in the EAM condition, the task requirement to lie about the secret date should instead focus attention on the one recent episodic memory to cue the task response, while also minimizing attention to any associated semantic memory or knowledge, because recent episodic memory most clearly distinguishes the secret date from the target date and irrelevants. Moreover, the EAM condition probably focuses attention on episodic memory more than the SAM condition because strategic retrieval processes in inferior prefrontal cortex are required more for remote than recent episodic recollection (Soderlund et al., 2012), and lying recruits such prefrontal processes to inhibit the prepotent truthful response. Altogether, this would make these neural resources less available to recollect episodic autobiographical memories, which is more of a problem for the birth date in the SAM condition where in these memories are more remote, than the EAM condition where in the memory is recent. Likewise, such prefrontal processes are also implicated in selecting episodic memory from competing alternatives (Badre and Wagner, 2007), and the many remote episodic memories associated with the birthdate would require such selection processes more than the single recent episodic memory associated with the secret date. Consequently, the SAM condition orients the task predominantly toward using semantic memory as the primary cue to guide performance because semantic memory is retrieved more readily than is remote episodic memory. In contrast, the EAM condition orients the task predominantly toward recent episodic memory as the primary cue to guide performance. This predicts that the SAM condition should produce larger CIT effects on ERP components related to knowledge and semantic memory (i.e., N2, N400, and P3b), whereas the EAM condition should produce larger CIT effects on ERP components related to episodic memory (i.e., LPC), consistent with these findings. Such task orientation effects would then interact with the memory retrieved to determine task performance (Schyns, 1998).

## Conclusions

This study demonstrates that memory associated with a probe has multiple effects on the ERPs in CIT paradigms (N2, N400, P3b, and LPC), and the exact pattern of each effect depends upon the type of memory. Here, these effects are broadly consistent with the known properties of semantic and episodic memory systems, as assessed using ERPs. Documenting and examining these ERP effects is necessary to understand fully the neural basis of the processes engaged during CIT and related paradigms. For practical applications, analysis methods may be fine-tuned to detect concealed information depending on the associated memory type: For semantic probes, the focus should be the frontal N2, centroparietal N400, and parietal P3b, and for episodic probes the focus should be the P3b, and the LPC. Further, using longer sessions with more trials can efficiently improve signal-to-noise ratio because ERP CIT effects exhibited no signs of habituation or fatigue.

Altogether, the findings indicate that the frontal N2, centroparietal N400, and P3b are especially sensitive to information stored in knowledge and semantic memory systems of the neocortex, whereas the LPC is especially sensitive to information stored in the episodic memory system that depends on the hippocampus and adjacent cortical structures of the mediotemporal lobe. This conclusion highlights that clearly defining, manipulating, and considering the type of memory that may be concealed may be important for accurate detection of concealed information. Future CIT studies will need to consider carefully the semantic and episodic memory associated with each item, as well as how this interacts with task and experimental context. For example, if the goal is to reveal concealed episodic memory, then the semantic memory associated with each episodic item may need to be equated or specifically manipulated to separate out the semantic from episodic contributions. The present memory manipulation essentially orients subjects to focus on one memory system over another because the semantic and episodic probes that determined the memory system (i.e., semantic vs. episodic) for retrieval were presented in separate blocks of trials; participants did not have to discriminate between semantic and episodic probes directly. The strength of this approach is that it parallels the methods of memory research. Semantic memory experiments would involve asking subjects to report if the item exists in the real world, the meaning of a stimulus, or to categorize or name it, and items would differ in how well they are known (i.e., how well semantic memory is activated), akin to the semantic probe and irrelevants in this CIT paradigm. Episodic memory experiments would involve asking subjects to report whether the item is familiar from a prior study experience and/or to recollect associated information from that study experience, and familiar items would be mixed with unfamiliar items that had not been studied, akin to the episodic probe and irrelevants in this CIT paradigm. The present experiment was a first attempt at teasing apart semantic and episodic memory contributions to the CIT. However, here, as in the real world, items will usually activate both semantic and episodic memory to some extent. This mix needs to be carefully documented in CIT paradigms and, more broadly, in deception research.

### Conflict of interest statement

The authors declare that the research was conducted in the absence of any commercial or financial relationships that could be construed as a potential conflict of interest.
